# Supplementary material for: Preoperative Immune Cell Dysregulation Accompanies Ovarian Cancer Patients into the Postoperative Period
Source: Int J Mol Sci. 2024 Jun 28;25(13):7087. doi: 10.3390/ijms25137087 (PMC11240929; doi:10.3390/ijms25137087)
Supplement: Supplementary file 1 [file ijms-25-07087-s001.zip › ijms-3045168-supplementary.pdf]

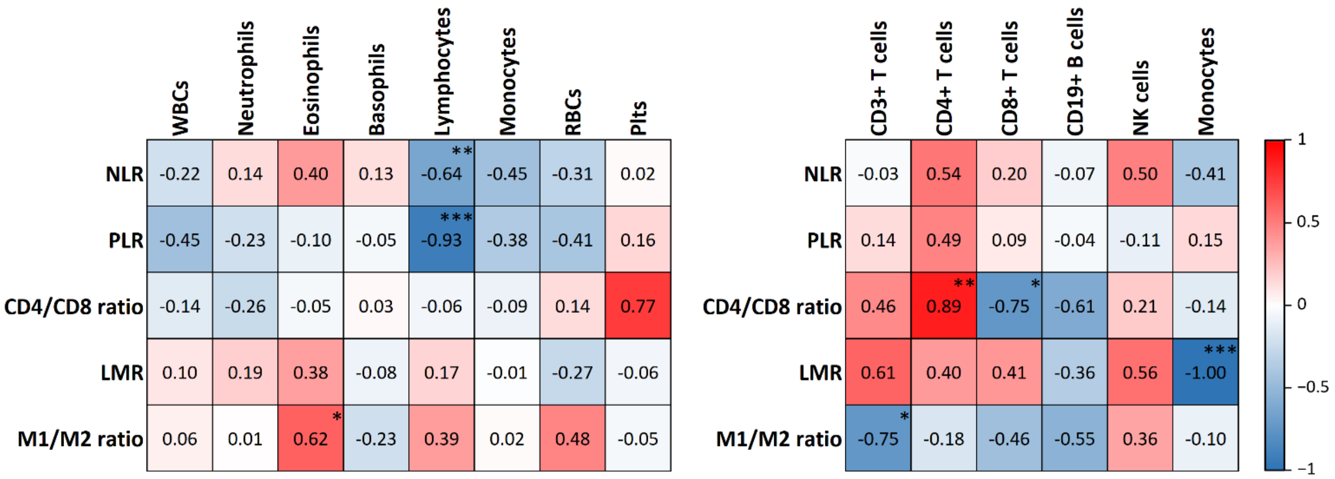


**Figure S1**. Correlation between peripheral blood cell ratios and peripheral blood cells in the control group. The left graph presents the correlation between the ratios and quantitative values of blood cell counts. The right graph illustrates the correlation between the ratios and proportional values of PBMCs among all PBMCs. The listed values indicate Spearman's correlation coefficient (rs). Colors in the graphs range from blue to red, indicating rs from −1 to 1, respectively. All p-values of correlations are >0.05 unless otherwise stated. * $p < 0.05$; ** $p \leq 0.01$; *** $p \leq 0.001$.
